# Suicidal Risk and Depression in Pregnant Women in Times of Pandemic

**DOI:** 10.1007/s10995-023-03688-3

**Published:** 2023-06-08

**Authors:** Mirta Solis, Mercedes Valverde-Barea, Luis Gutiérrez-Rojas, Inmaculada Romera, Sheila Cruz-Bailén, Sara Jiménez-Fernández

**Affiliations:** 1 Child and Adolescent Mental Health Unit, Jaén Medical Center, Jaén, Spain; 2 Department of Psychiatry and CTS-549 Research Group, Institute of Neurosciences, University of Granada, Granada, Spain; 3 Psychiatry Service, Hospital Universitario San Cecilio, Granada, Spain; 4 Psychiatry Service, Hospital Universitario Virgen de la Victoria, Málaga, Spain

**Keywords:** Pregnancy, Suicide, Depression, Early detection

## Abstract

**Purpose:**

Pregnancy is a risk period for the development of mental disorders. About 10% of pregnant women worldwide experience a mental disorder, mainly depression, and this percentage has been aggravated by the COVID-19 pandemic. This study aims to understand the impact of COVID-19 on the mental health of pregnant women.

**Methods:**

Three hundred and one pregnant women in the week 21.85 ± 9.9 were recruited through social media and pregnant women forums from September 2020 to December 2020. A multiple-choice questionnaire was administered to evaluate the sociodemographic characteristics of the women, the care provided, and different aspects related to COVID-19. A Beck Depression Inventory was also delivered.

**Results:**

Of the pregnant women 23.5% had seen or had considered seeing a mental health professional during pregnancy. Predictive models using multivariate logistic regression found that this fact was associated with an increased risk of depression (OR = 4.22; CI 95% 2.39–7.52; *P* < 0.001). Among women with moderate-severe depression, it was associated with an increased risk of having suicidal thoughts (OR = 4.99; CI 95% 1.11–27.9; *P* = 0.044) and age was found to be a protective variable (OR = 0.86; CI 95% 0.72–0.98; *P* = 0.053).

**Conclusions:**

The COVID-19 pandemic represents a major mental health challenge for pregnant women. Despite the decrease in face-to-face visits, there are opportunities for health professionals to identify the existence of psycho-pathological alterations and suicidal ideation by asking the patient if she is seeing or considering seeing a mental health professional. Therefore, it is necessary to develop tools for early identification to ensure correct detection and care.

## Significance

Among women with moderate-severe depression, considered seeing a mental health professional during pregnancy was associated with an increased risk of having suicidal thoughts and age was found to be a protective variable. Despite the decrease in face-to-face visits during COVID-19 pandemic, there are opportunities for health professionals to identify the existence of psycho-pathological alterations and suicidal ideation by asking the patient if she is seeing or considering seeing a mental health professional. Asking about their intention to seek specialized assistance can help to identify those women at risk of suffering affective disorders such as depression and suicidal ideation and to individually plan future medical visits.

## Introduction

The new 2019 coronavirus (COVID-19), first described in Wuhan, Hubei Province, China, in late 2019, is a disease with high infectivity and spreading power. The World Health Organization (WHO) soon acknowledged it as a global public health emergency (Cucinotta & Vanelli, 2020). As a consequence, the care of pregnant women was also transformed. Some of the most important challenges for gynecologists and obstetrics units faced with the pandemic have been following up patients during confinement, being able to coordinate complementary exams, and making pre-surgery appointments to minimize the presence of patients in the hospital (Luna Tomás et al., 2020; Riecher-Rössler 2022).

COVID-19 has brought with it several challenges: the implementation of methods to prevent transmission to pregnant women and their close environments; the adequacy of birth preparation in positive cases; the prevention of maternal stress and emotional exhaustion; appropriate newborn care, and evaluation of new protocols (López, 2020). Professionals have had to adapt to telematic assistance in order to provide security and prevent transmission, combining consultations to avoid the need for pregnant woman to travel so often (Baena-Antequera et al., 2020).

In addition, pandemics have important psychological effects on the population, derived from the perception of uncertainty, confusion, and sense of urgency that they generate (Nalbandian et al., 2021), just as covid-19 has done (Ahorsu et al., 2020; Quintero et al., 2020). Women are often more fearful of infection and the increased risk of mental health problems after the occurrence of stressful life events than men are, due to differences in sensitivity and susceptibility to stress (Maunder et al., 2003; Tzur Bitan et al., 2020).

Pregnancy and the transition to motherhood involve major psychological and social changes in the mother, which have been related to the presence of symptoms of anxiety and depression (Teixeira et al., 2009). On the other hand, the occurrence of stressful events during pregnancy, such as the death of a family member, exposure to natural disasters or the outbreak of a pandemic that generates interpersonal imbalances could increase concerns during pregnancy and affect the mental health of pregnant women (Holditch-Davis et al., 2007). Therefore, it is recommended to pay special attention to the presence of fears and identify mental pathologies in pregnant women during the pandemic (Salehi et al., 2020).

Depression affects approximately 20% of women throughout life, with pregnancy being a period of high vulnerability (Rich-Edwards et al., 2006). Depression is the most common psychiatric disorder during pregnancy, with a prevalence ranging from 4 to 25% (Faisal-Cury & Rossi, 2007).

Depression during pregnancy is an important issue of public health, due to three main reasons: (1) it is the strongest risk factor for presenting postpartum depression (Heron et al., 2004) and (2) leads to adverse maternal and fetal outcomes (Bansil et al., 2020). Also, stress during pregnancy has been found to be positively correlated with the risk of exhibiting anxiety and depression (Tang et al., 2019). Therefore, psychopathological assessment during pregnancy is essential to detect pregnant women in need of intervention in order to safeguard the well-being of both the mother and the baby (Ajinkya et al., 2013). During the COVID-19 pandemic, pregnant women have presented more psychiatric symptoms than those assessed before the pandemic, mainly in the form of depressive disorders and symptoms of anxiety (Berthelot et al., 2020). However, the presence of autolytic ideation has not been evaluated nor have any mechanisms of this been explored. Against the backdrop of events such as COVID-19, we must recognize the potential for adverse perinatal mental health consequences to be of critical public health concern, and provide patients with care and support to prevent and ameliorate their negative effects (Shoib et al., 2020).

The goal of this study is to evaluate the impact of the COVID-19 pandemic on the mental health of pregnant patients contacted through social media networks during the first few months of the pandemic, and to identify protective and risk factors for the exhibition of depressive symptoms and to assess the presence of suicidal ideation.

## Material and Methods

### Study Design and Population

The present study was conducted between September 2020 and December 2020, during the state of alert due to the COVID-19 crisis. It is an observational study made up of 301 pregnant women recruited through social media networks (Facebook and WhatsApp) and Spanish-language pregnant women forums (WOOM App and Menstrual Calendar App.). All of them completed an anonymous and voluntary online survey using the online tool “Google Forms”.

The survey included multiple-choice questions for the assessment of sociodemographic aspects: age (in years), country of residence, week of gestation and marital status. It also asked whether they had considered the need to see a mental health professional such as a psychologist or psychiatrist in the last month; whether primary care physicians and/or nurses (midwives) had inquired about their mental health; if they had any concerns or fears about becoming infected and having to be isolated; whether they were afraid of becoming infected by COVID-19 and causing harm to the fetus; and whether they were afraid that other people were experiencing active COVID-19 infection and were not taking the appropriate protective or isolation measures.

Clinical depression was also assessed using the Beck Depression Inventory (BDI) (Beck et al., 1996). The BDI consists of 21 items, each with 4 alternative responses, which evaluate the intensity of the symptom. The questionnaire used is one of the most quoted in literature and has predictive validity as a diagnostic screening tool in the general population with a sensitivity of 100%, specificity of 99%, positive predictive value of 0.72 and negative predictive value of 1. The results are classified as: minimal symptoms of depression < 13 points, mild depression 14–18 points, moderate depression 19–27 points and severe depression 28–64 points.

The study was carried out in accordance with the 1975 Declaration of Helsinki. The data were processed in accordance with the provisions of Act 3/2018, (on December 5th), under the Protection of Personal Data and Guarantee of Digital Rights and approved by the Ethics Committee of the Andalusian Health Service.

## Statistical Analysis

The sample was described by descriptive analysis using mean and standard deviation (for continuous quantitative variables) and frequencies (in case of categorical variables).

For the comparison between groups, the *t*-Student test was used in the case of continuous variables and the Chi-square test for qualitative or categorical variable. In this case, the total score of the BDI (and the different symptoms it includes) was compared to the trimester of pregnancy at the time of the survey, whether the mother was part of the single-parent group, and whether the health care providers in charge of the mother’s periodic controls inquired about her mental health status.

Lastly, three predictive models were created using multivariate logistic regression for which the dependent variables “depression”, “irritability” and “suicidal ideas” were used. “Depression” (used according to the BDI total score), and “irritability” (from item 11 of the BDI) to assess the presence and severity of irritability (Pedrelli et al., 2013). So “irritability” was considered when the values scored mild-moderate-severe and “no depression” or “no irritability” when they scored less than mild. In addition, “suicidal ideas” (from item 9 of the BDI) was used to assess the presence and severity of suicidal ideas (Casey et al., 2008; Rangel-Garzón et al., 2015). Pedrelli et al., used item 11 of the BDI separately and found a statistically significant relationship between symptoms of depression and irritability and warned that individuals with severe irritability presented greater intensity of depressive symptoms than those without irritability or moderate irritability (Pedrelli et al., 2013). The use of a single item suicide or a dimensional factor derived from a depression scale might be a valid approach to assess suicidal ideations (Desseilles et al., 2012). Sociodemographic variables were included in the model, as well as the perception variables of the pregnant women in relation to their psychological state (need for help) and the COVID19 pandemic (fear of becoming infected, affecting the baby, satisfaction with their care). Non-significant variables were extracted from the initial model by the “backward” method. Possible interactions were analyzed. In the predictive model “depression” one interaction was detected and two new ones were performed according to whether the patients had “severe” or “mild-moderate” depression. The level of statistical significance was set at 0.05. All analyses were performed using the R commander 5.3 statistical package.

## Results

In this observational study, a sample was obtained that included 301 pregnant patients. Social and clinical characteristics are summarized in Table 1

**Table 1:** Sociodemographic and medical characteristics of participants

Characteristics	Total [*n* = 301 (100%)]
Age, years (mean ± SD)	32 ± 4.66
Week of pregnancy (mean ± SD)	21.85 ± 9.9
*Trimester of pregnancy*	
1º Trimester, *n* (%)	71 (23.6%)
2º Trimester, *n* (%)	135 (45%)
3º Trimester, *n* (%)	95 (31.5%)
*Residency*	
Spain, *n* (%)	262(87%)
Other countries, *n* (%)	39 (13%)
*Type of family*	
Single parent, *n* (%)	33 (11%)
Biparental, *n* (%)	268 (89%)
*Thinking about or being under Mental Health treatment*	
Yes, *n* (%)	71(23.5%)
No, *n* (%)	230(76.5%)
*Interviewed about her mental health status by care providers*	
Si, *n* (%)	45(15%)
No, *n* (%)	256(85%)
*Worried if COVID-19 patients follow recommendations*	
Si, *n* (%)	272(90.3%)
No, *n* (%)	29 (9.7%)
*Beck Depression Inventory (me*an ± *SD)*	13.53 ± 8.67
No depression, *n* (%)	172 (57,14%)
Mild depression, *n* (%)	51 (16.94%)
Moderate depression, *n* (%)	57(18.93%)
Severe depression, *n* (%)	21 (6.97%)
*Age of women with severe symptoms (me*an ± *SD)*	33.85 ± 5.88

Statistically significant differences were found in the total score of the BDI according to whether the pregnant women were thinking of consulting a psychiatrist/psychologist (not attending/not thinking of attending 11.69 ± 7.16 vs. attending/thinking of attending 19.52 ± 10.34; *P* < 0.001). But no/very little difference was found in the BDI score according to the trimester of gestation (first trimester 12.90 ± 9.69; second trimester 13.69 ± 8.48; third trimester 13.79 ± 8.19; *P* = 0.779), type of cohabitation (single parent 15.61 ± 11.30 vs. couple 13.28 ± 8.29; *P* = 0.146), having been interviewed about their mental health (not having been interviewed 13.63 ± 8.47 vs. yes 13.00 ± 9.83; *P* = 0.654), experiencing concern about being with positive people (not concerned 13.15 ± 8.09 vs. concerned 13.61 ± 8.79; *P* = 0.74) or about being infected and having to isolate themselves (not concerned 13.64 ± 8.58 vs. concerned 13.53 ± 8.69; *P* = 0.97).

In the predictive model we found that, in pregnant women, thinking about going to a consultation/going to a consultation with a mental health professional increased the risk of having depressive symptoms (F 1.44; OR = 4.22; CI 95% 2.39–7.52; *P* < 0.001). None of the other variables included in the model predicted the risk of presenting depression (Table 2).

**Table 2:** Predictive models of depressive symptoms (A) and suicidal ideation (B)

(A) Depressive symptoms	*Z*	*Crude OR*	*Adjusted OR*	*p*
Age	0.522	< 0.001	1.015	0.602
Attending	4.937	4.23	4.22	** < 0.001**

(B) Suicidal ideation				
BDI score	3.928	1.11	1.11	** < 0.0001**
Age	− 1.927	0.95	0.90	0.054
Single parent family	− 1.643	0.72	0.18	0.100
Concern about being infected (yes)	1.337	2.13	3.31	0.181
Concern about being with a positive (yes)	− 1.815	0.72	0.24	0.0696
Attending a consultancy	2.237	5.40	3.14	**0.0253**

The significance of bold is *p* < 0.05

**Fig. 1:**
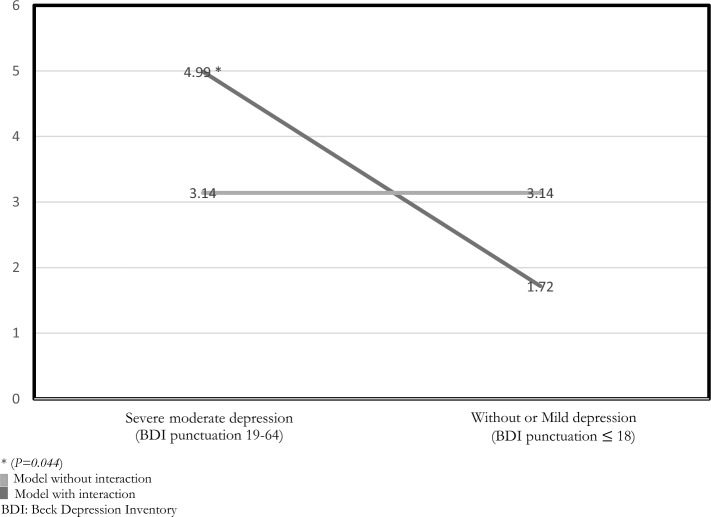
Interaction between BDI punctuation and thinking about going to a consultation with a mental health provider (in the statistical predictive model)

A predictive model was also designed for suicidal ideation. In this case, an interaction was identified between the BDI score and thinking about requesting mental health consultation (Fig. 1). According to the degree of depression (mild depression or moderate-severe depression), it was observed that age reduced the risk of suicidal ideation appearing in women with moderate-severe depression (F = -0.153; OR = 0.86; CI 95% 0.72–0.98; *P* = 0.053), while a total score on the BDI (F = 0.132; OR = 1.14; CI 95% 1.04–1.28; *P* = 0.006) and thinking about going to a consultation (F = 1.607; OR = 4.99; CI 95% 1.11–27.9; *P* = 0.044) significantly increased the risk of suicidal ideas. However, these variables did not prove to be predictors of suicidal ideation in pregnant women with mild depression.

## Discussion

In our research we have found that 26% of the pregnant women had moderate or severe depression. However, only 15% of the women had been interviewed about these aspects during the pandemic. Women who thought of consulting a psychiatrist had scored higher in depressive symptoms, and an affirmative answer would increase the risk of depression by a factor of 4. Regarding suicidal ideation, we found that age reduced the risk of suicidal ideation in women with moderate-severe depression whereas thinking about going to a consultation significantly increased the risk.

We found a prevalence of depression ranging from 15 to 21%. A 2016 review of fifteen included studies found the combined prevalence during the prenatal period to be 17.2% (Underwood et al., 2016). However, all of the included studies relied on prevalence from self-report symptom scales, rather than diagnostic instruments, which may overestimate the true prevalence (Gavin et al., 2005). Another review of perinatal depression in low- and middle-income countries by Gelaye et al. found the prevalence of prenatal depression to be at 25.8% (Gelaye et al., 2016). Woody et al. responds to these gaps in literature by summarizing current available literature on the prevalence of perinatal depression (Woody et al., 2017).

Among the factors identified that affect the mental health of pregnant women are women’s concern about the health of the fetus and the outcome of delivery (Bayrampour et al., 2015). In our study, almost the entire sample (96%) claimed to be afraid of getting infected by COVID-19 and potentially affecting their baby’s health.

During the pandemic, another point of difficulty has been the periodic in-person gynecological controls in hospitals and health centers. 84.3% of pregnant women expressed a fear of being infected by COVID-19. It has been noted that, during pregnancy, mothers suffering from infectious diseases are more concerned about the health of their baby than the control group without infectious diseases (Khorsandi et al., 2016). A characteristic element of infectious diseases compared to other conditions is the fear of contagion (Ahorsu et al., 2020).

During the COVID-19 pandemic, as well as the fear of the baby becoming infected, concern has been raised about the possible consequences of repeated use of disinfectants because of the potential injurious risk to the fetus (Fakari & Simbar, 2020). Pregnant women also expressed a fear of infecting loved ones or others as a generator of anxious symptomatology (Colizzi et al., 2020). 84.3% of the members of our study expressed concern about becoming infected and having to be isolated or confined, and the vast majority (90.3%) reported concern that COVID-19 positive patients would not have adequate protective measures, would not comply with confinement properly, and could jeopardise the health of their baby. In a study conducted on an Iranian Hospital in September 2020, 222 pregnant women found that anxiety during pregnancy correlated positively with mental problems. The authors proposed that during the pandemic period, special attention should be paid to the fears of pregnant women in order to pre-identify any mental disorders that may appear (Salehi et al., 2020).

It is important to note that depressive symptoms are commonly under-diagnosed in pregnant women and one of the reasons may be that they can be confused with the usual symptoms of pregnancy such as sleep disturbances (mainly insomnia), appetite fluctuations, loss of energy, fatigue and changes in libido However, in our study, only 15% of the pregnant women interviewed had been asked by their midwives or physicians about aspects of their mental health status during the pandemic time. This could lead to under-diagnosis of affective disorders in this population, which in themselves are commonly underdiagnosed because of pregnancy such as sleep disturbances (mainly insomnia), appetite fluctuations, loss of energy, fatigue, and changes in libido (Clark et al., 2020).

We also found that the thought of consulting a mental health specialist multiplied the risk of presenting suicidal ideas in women with moderate-severe symptoms by five. According to the literature, most pregnant women who committed suicide had more psychiatric comorbidities than pregnant control patients who tried to commit suicide and failed (Grigoriadis et al., 2017). This fact suggests that many of the women at risk could have been identified from their prior mental health history. According to The Joint Commission’s 2016 database of 1089 reports of suicide in the general population between 2010 and 2014, deficiencies in psychopathological assessment of the patient were the most frequently cited cause (The Joint Commission, 2016).

Primary care professionals have the closest relationship with patients, and because of this, when patients experience mood changes or stressors appear that may precipitate suicidal behaviors, they are more likely to seek help from these professionals (Harmer et al., 2021). However, 85% of pregnant respondents claimed not to have been examined or questioned by their referral health team at their regular consultations and medical controls about their mental health status during the pandemic.

In our sample we also observed that age is a protective factor for suicidal risk (OR = 0.86) among those women with a high score on the BDI scale (moderate-severe depression). These results seem to coincide with previously published results. Kim and col. found in a systematic review that suicidal ideation is positively associated with **a** younger maternal age (Kim et al., 2015). In our population, we also found that thinking about seeing a mental health professional multiplied the risk of presenting suicidal ideation by a factor of 5 (OR = 4.99) in the group with moderate-severe symptomatology; in this group, age behaves as a protective factor. Others researchers have found an association between younger age and depression in pregnant adolescents, perhaps because these studies have been done in developing countries (Oladeji et al., 2022).

Less than half of the women who died by suicide during pregnancy or postpartum had used mental health services in their last month of life (Grigoriadis et al., 2017). Unlike the general population, pregnant women are regularly screened by health professionals, which provides greater opportunities for screening (Arachchi et al., 2019).

When considered in its entirety, the evidence suggests that pregnant women are more likely than the general population to experience suicidal ideation (Newport et al., 2007), but less likely than their non-pregnant counterparts to commit suicide (Gissler et al., 2005). The reported prevalence of suicidal ideation ranges from 3 to 33% (Gentile, 2011). In our population, nearly 8% of pregnant women reported having some suicidal thoughts during gestation, but felt they would not be able to carry it out. Of these, most had a BDI score of major depression.

Suicidal behavior in the postpartum period (including suicidal ideation, attempts and completed suicide) is very often preceded by suicidal behavior before delivery (Lindahl et al., 2005; Nock et al., 2008). This pattern of antepartum conditions being associated with postpartum risk is common in mood disorders such as anxiety and stress (Gavin et al., 2011). Thus, the antepartum period represents a critical time and opportunity for early identification.

### Strengths and Limitations

One of the main strengths of our study is the significant sample size, with a total number of 301 participants. Another one is the objective itself, which is to identify women at risk of presenting a mental problem at a time when contact is limited and time is scarce, and these factors can be transformed into a screening tool. The fact that the women were not selected from hospitals makes it possible to minimize selection bias.

On the other hand, this fact also confers greater heterogeneity to the sample since they are women residing in different parts of the world whose socioeconomic circumstances are unknown, a variable which has not been studied and which carries a lot of weight in mental health. Another limitation of this study lies in its design; as it is a cross-sectional study, it was not possible to follow up this group of patients or to monitor their mental state after childbirth.

## Conclusions

The COVID-19 pandemic represents a great challenge for the mental health of pregnant women. Despite the limited number of consultations, there are multiple opportunities for health professionals to identify the existence of psychopathological disorders and the presence of suicidal ideation. For this reason, the development of tools for early identification, either in person or online, is necessary to ensure individualized care for pregnant women.

Asking about their intention to seek specialized assistance can help to identify those women at risk of suffering affective disorders such as depression and suicidal ideation and to individually plan future medical visits.

## References

[CR1] Ahorsu DK, Lin CY, Imani V, Saffari M, Griffiths MD, Pakpour AH (2020). The fear of COVID-19 Scale: Development and initial validation. International Journal of Mental Health and Addiction..

[CR2] Ajinkya S, Jadhav PR, Srivastava NN (2013). Depression during pregnancy: Prevalence and obstetric risk factors among pregnant women attending a tertiary care hospital in Navi Mumbai. Industrial Psychiatry Journal..

[CR3] Arachchi NSM, Ganegama R, Husna AWF, Chandima DL, Hettigama N, Premadasa J, Herath J, Ranaweera H, Agampodi TC, Agampodi SB (2019). Suicidal ideation and intentional self-harm in pregnancy as a neglected agenda in maternal health; an experience from rural Sri Lanka. Reproductive Health..

[CR4] Baena-Antequera F, Jurado-García E, Fernández-Carrasco FJ, Rodríguez-Díaz L, Gómez-Salgado J, Vázquez-Lara JM (2020). Pregnancy care during COVID-19 epidemic, a drive for change?. Revista Española De Salud Publica..

[CR5] Bansil P, Kuklina EV, Meikle SF, Posner SF, Kourtis AP, Ellington SR, Jamieson DJ (2020). Maternal and fetal outcomes among women with depression. Journal of Women’s Health..

[CR6] Bayrampour H, McDonald S, Tough S (2015). Risk factors of transient and persistent anxiety during pregnancy. Midwifery.

[CR7] Beck AT, Steer RA, Brown GK (1996). BDI-II: Beck depression inventory.

[CR8] Berthelot N, Lemieux R, Garon-Bissonnette J, Drouin-Maziade C, Martel É, Maziade M (2020). Uptrend in distress and psychiatric symptomatology in pregnant women during the coronavirus disease 2019 pandemic. Acta Obstetricia Et Gynecologica Scandinavica..

[CR9] Casey P, Dunn G, Kelly BD, Lehtinen V, Dalgard OS, Dowrick C, Ayuso-Mateos JL (2008). The prevalence of suicidal ideation in the general population: Results from the outcome of depression international network (ODIN) study. Social Psychiatry and Psychiatric Epidemiology..

[CR10] Clark CT (2020). Psychotropic drug use in perinatal women with bipolar disorder. Seminars in Perinatology..

[CR11] Colizzi M, Bortoletto R, Silvestri M, Mondini F, Puttini E, Cainelli C (2020). Medically unexplained symptoms in the times of COVID-19 pandemic: A case-report. Brain, Behavior and Immunity-Health..

[CR12] Cucinotta D, Vanelli M (2020). WHO declares COVID-19 a pandemic. Acta Biomedica..

[CR13] Desseilles M, Perroud N, Guillaume S, Jaussent I, Genty C, Malafosse A, Courtet P (2012). Is it valid to measure suicidal ideation by depression rating scales?. Journal of Affective Disorders..

[CR14] Faisal-Cury A, Rossi Menezes P (2007). Prevalence of anxiety and depression during pregnancy in a private setting sample. Archives of Women’s Mental Health..

[CR15] Fakari FR, Simbar M (2020). Coronavirus pandemic and worries during pregnancy; a letter to editor. Archives of Academic Emergency Medicine..

[CR16] Gavin AR, Tabb KM, Melville JL, Guo Y, Katon W (2011). Prevalence and correlates of suicidal ideation during pregnancy. Archives of Women’s Mental Health..

[CR17] Gavin NI, Gaynes BN, Lohr KN, Meltzer-Brody S, Gartlehner G, Swinson T (2005). Perinatal depression: A systematic review of prevalence and incidence. Obstetrics and Gynecology..

[CR18] Gelaye B, Rondon MB, Araya R, Williams MA (2016). Epidemiology of maternal depression, risk factors, and child outcomes in low-income and middle-income countries. Lancet Psychiatry.

[CR19] Gentile S (2011). Suicidal mothers. Journal of Injury and Violence Research..

[CR20] Gissler M, Berg C, Bouvier-Colle MH, Buekens P (2005). Injury deaths, suicides and homicides associated with pregnancy, Finland 1987–2000. European Journal of Public Health..

[CR21] Grigoriadis S, Wilton AS, Kurdyak PA, Rhodes AE, VonderPorten EH, Levitt A (2017). Perinatal suicide in Ontario, Canada: A 15-year population-based study. Canadian Medical Association Journal..

[CR22] Harmer B, Lee S, Doung TH, Saadabadi A.(2021). Suicidal Ideation. Ed. Treasure Island, FL: StatPearls Publishing 33351435

[CR23] Heron J, O’Connor TG, Evans J, Golding J, Glover V, ALSPAC Study Team (2004). The course of anxiety and depression through pregnancy and the postpartum in a community sample. Journal of Affective Disorders..

[CR24] Holditch-Davis D, Santos H, Levy J, White-Traut R, O’Shea TM, Geraldo V, David R (2007). Patterns of psychological distress in mothers of preterm infants. Infant Behavior and Development..

[CR25] Khorsandi M, Vakilian K, Salehi B, Goudarzi MT, Abdi M (2016). The effects of stress inoculation training on perceived stress in pregnant women. Journal of Health Psychology..

[CR26] Kim JJ, La Porte LM, Saleh MP, Allweiss S, Adams MG, Zhou Y, Silver RK (2015). Suicide risk among perinatal women who report thoughts of self-harm on depression screens. Obstetrics & Gynecology..

[CR27] Lindahl V, Pearson JL, Colpe L (2005). Prevalence of suicidality during pregnancy and the postpartum. Archives of Women’s Mental Health.

[CR28] López OP (2020). Especificidades, prioridades y desafíos para el rol de la matrona, matrón, en contexto de pandemia por COVID-19. Revista Matronería Actual..

[CR29] LunaTomás MA, MargelíVila M, Gozálvez R (2020). Influence of the 2019-novel coronavirus pandemic on the management of breast cancer. Clinica en Investigación en Ginecologia y Obstetricia.

[CR30] Maunder R, Hunter J, Vincent L, Bennett J, Peladeau N, Leszcz M (2003). The immediate psychological and occupational impact of the 2003 SARS outbreak in a teaching hospital. Canadian Medical Association Journal..

[CR31] Nalbandian A, Sehgal K, Gupta A (2021). Post-acute COVID-19 syndrome. Nature Medicine..

[CR32] Newport DJ, Levey LC, Pennell PB, Ragan K, Stowe ZN (2007). Suicidal ideation in pregnancy: assessment and clinical implications. Archive of Women’s Mental Health..

[CR33] Nock MK, Borges G, Bromet EJ, Cha CB, Kessler RC, Lee S (2008). Suicide and suicidal behavior. Epidemiologic Reviews..

[CR34] Oladeji BD, Bello T, Ayinde O, Idowu P, Gureje O (2022). Prevalence and correlates of depression among pregnant adolescents in primary maternal care in Nigeria. Archives of Women’s Mental Health..

[CR35] Pedrelli P, Nyer M, Holt D, Bakow BR, Fava M, Baer L, Cassiello C, Mulligan M, Cusin C, Farabaugh A (2013). Correlates of irritability in college students with depressive symptoms. Journal Nervous and Mental Diseases.

[CR36] Quintero J, Mora F, Rodríguez-Quiroga A, de Alvarez MMÁ, López-Ibor MI (2020). Post-COVID mental health. Actas Españolas de Psiquiatría..

[CR37] Rangel-Garzón C, Suárez-Beltrán M, Escobar-Córdoba F (2015). Risk suicide assessment scales in primary care. Revista De La Facultad De Medicina, Universidad Nacional De Colombia.

[CR38] Rich-Edwards JW, Kleinman K, Abrams A, Harlow BL, McLaughlin TJ, Joffe H, Gillman MW (2006). Sociodemographic predictors of antenatal and postpartum depressive symptoms among women in a medical group practice. Journal of Epidemiology and Community Health..

[CR39] Riecher-Rössler A (2022). Editorial: women and the pandemic. Archives of Women’s Mental Health..

[CR40] Salehi L, Rahimzadeh M, Molaei E, Zaheri H, Esmaelzadeh-Saeieh S (2020). The relationship among fear and anxiety of COVID-19, pregnancy experience, and mental health disorder in pregnant women: A structural equation model. Brain and Behavior..

[CR41] Shoib S, Arafat SMY, Ahmad W (2020). Perinatal mental health in Kashmir, India during the COVID-19 pandemic. Maternal and Child Health Journal..

[CR42] Tang X, Lu Z, Hu D, Zhong X (2019). Influencing factors for prenatal stress, anxiety and depression in early pregnancy among women in Chongqing, China. Journal of Affective Disorders..

[CR43] Teixeira C, Figueiredo B, Conde A, Pacheco A, Costa R (2009). Anxiety and depression during pregnancy in women and men. Journal of Affective Disorders..

[CR44] The Joint Commission (TJC). (2016). Sounding the alarm about suicide risk. ED Management. *28* (5), 49–54. 27265999

[CR45] TzurBitan D, Grossman-Giron A, Bloch Y, Mayer Y, Shiffman N, Mendlovic S (2020). Fear of COVID-19 scale: Psychometric characteristics, reliability and validity in the Israeli population. Psychiatry Research..

[CR46] Underwood L, Waldie K, D’Souza S, Peterson ER, Morton S (2016). A review of longitudinal studies on antenatal and postnatal depression. Archives of Women’s Mental Health..

[CR47] Woody CA, Ferrari AJ, Siskind DJ, Whiteford HA, Harris MG (2017). A systematic review and meta-regression of the prevalence and incidence of perinatal depression. Journal of Affective Disorders..

